# The effectiveness of cognitive behavior stress management on functional dyspepsia symptoms

**Published:** 2015-04

**Authors:** ZAHRA DEHGHANIZADE, YADOLAH ZARGAR, MAHNAZ MEHRABIZADEH HONARMAND, AHMAD KADKHODAIE, MAJID EYDI BAYGI

**Affiliations:** 1 Young Researchers and Elite Club, Islamic Azad University, Shiraz Branch, Shiraz, Iran;; 2 Department of Psychology and Educational Sciences, Shahid Chamran University, Ahvaz, Iran;; 3 Department of Psychology and Educational Sciences, Shahid Chamran University, Ahvaz, Iran;; 4 Ahvaz University of Medical Sciences, Ahvaz, Iran;; 5 Young Researchers and Elite Club, Islamic Azad University, Torbate Heydarie Branch, Torbate Heydarie, Iran;

**Keywords:** Cognitive behavior, Dyspepsia, Stress

## Abstract

**Introduction:**

Functional dyspepsia and digestive disorders are common, debilitating and costly. Little information is available about the role of stress management in terms of cognitive-behavioral treatment of dyspepsia. The purpose of this study was to investigate the effectiveness of cognitive-behavioral stress management on functional dyspepsia symptoms in patients who referred to digestion clinics in Ahvaz.

**Methods:**

This was a quasi-experimental study, using pre-test, post-test and follow-up with control group. The sample size was decided according to availability. For this purpose, 30 patients were selected using Rome-III diagnostic criteria with diagnosis of functional dyspepsia. They were divided with block randomization into two experimental and control groups (Each with 15 subjects). The experimental group received 10 sessions of cognitive behavioral stress management. But, the control group did not receive any intervention. Two groups were assessed at post-test and follow-up after the intervention. Data collection in this study was based on the NDI index. All subjects completed the NDI index for evaluating dyspeptic symptoms in the pre-test phase. At the end of interventions and post-test phase, both groups completed the NDI index. Also, all subjects completed a month after the end of the the NDI index in order to follow the effects of treatment. After the follow-up, the data were analyzed using the SPSS 14 and univariate analysis of variance. The significance level was considered p<0.05.

**Results:**

The result of this study showed that there were significant differences between the experimental and control groups in terms of functional dyspepsia in the post-test (p=0.000). Also, there were significant differences between the experimental and control groups in terms of functional dyspepsia in the follow-up (p =0.000).

**Conclusion:**

The results of the present study suggest that cognitive-behavioral stress management strategies are effective in reducing symptoms in patients with functional dyspepsia. So, cooperation among gastroenterologists, psychologists and psychiatrists can have positive effects on these patients.

## Introduction

Functional gastrointestinal disorders such as functional dyspepsia are not life threatening. This disease has an important role in daily activities, social activities and individual psychological conditions (1, 2). Functional dyspepsia is a symptomatic chronic disorder of the upper gastrointestinal tract which is not considered organic or biochemical abnormality. The symptoms include pain or discomfort in the upper abdomen, early satiety after eating, stomach fullness, bloating stomach, loss of appetite, frequent belching and nausea and there is no endoscopic evidence of an ulcer (3-5). The prevalence of dyspepsia in developing countries is estimated at 15 to 30 percent (6). Although the course of functional dyspepsia is not associated with mortality, ultimately an important issue is that this disease typically affects the lives of patients and the associated economic costs of care in the community (7-10). On the other hand, functional dyspepsia is a kind of psychosomatic disorder and some researchers have confirmed stable somatization in these patients (11, 12). Somatization functional gastrointestinal disorders are accompanied with psychiatric disorders (13-15). Thus, considering the role of psychological and social factors and the accompanied symptoms of psychological disorders in patients with functional dyspepsia, researchers have become interested in the use of complementary therapies, i.e. alternative and psychological (16). Lee et al. (2010) in their study found that individual symptoms associated with gastric motor disorders and visceral sensitivity can be affected by psychological stressors and may lead to functional dyspepsia (8). Therefore, extensive interventions such as psychotherapy or cognitive therapy may be effective in reducing symptoms of dyspepsia in patients with functional dyspepsia. Bagherian Sararudi et al. (2009) in research showed that the combination of psychological treatment based on stress management and typically medical treatment is a successful approach in treatment of patients with functional dyspepsia (17).

Cognitive behavior stress management refers to a family of stress management therapy which focuses on cognitive-behavioral approach. Stress management increases the ability of individuals to reduce stress and adaptability consistent with stressful situations (18). Mahadeva and Goh (2011) in their study investigated anxiety, depression and health-related quality of life in 839 patients with functional dyspepsia and indigestion physical. The results showed that health-related quality of life scores were lower in patients with functional dyspepsia compared with those with dyspepsia and toxin (19). Stress is an important factor affecting the symptoms of patients with functional dyspepsia; on the other hand, stress management increases the ability of people to reduce stress and appropriate compatibility with stressful situations. Therefore, the aim of this study was to investigate the effectiveness of cognitive-behavioral stress management on functional dyspepsia symptoms in patients who referred to digestion clinics in Ahvaz.

## Methods

This research was a quasi-experimental study, using with pre-test, post-test and follow-up with a control group. The research population consisted of all gastrointestinal patients referred to peptic clinic in Ahvaz city. After biochemical, endoscopy and ultrasound reviews, a gastroenterologist ruled out any kind of digestive organ disease and reached a diagnosis of functional dyspepsia. The samples were selected based on availability. For this purpose, 30 patients with diagnosis of functional dyspepsia, aged 20 to 50 years old, and willing to participate in the project were selected as sample by Rome-III diagnostic criteria. They were divided therough block randomization into two experimental and control groups (Each group of 15 subjects). Data collection in this study was based on the NDI index. All subjects completed the NDI index for evaluating dyspeptic symptoms in the pre-test phase. At the end of the interventions and post-test phase, both groups completed the NDI index. Also, all subjects completed the NDI index in order to follow the effects of treatment a month after the end of the study. After the follow-up, the data were analyzed in the SPSS 14, using univariate analysis of variance. Inclusion criteria for the study were normal upper endoscopy, normal abdominal sonography, and negative pylori test. Also, exclusion criteria included detecting any physical illness related symptoms during the study and using other psychological therapy during the study.

The present study used NDI index to evaluate symptoms of functional dyspepsia in patients. This index was constructed by Talley et al. (1999) to assess symptoms of functional dyspepsia (20). The reliability of this index was obtained using Cronbach's alpha coefficient in the range from 0.70 to 0.76 and its validity was measured using correlation coefficient; it was reported 0.83 to 0.71 (21). In the study of Tabib et al., validity and reliability of this index were reported favorable (22). In the present study, the reliability of this index using Cronbach's alpha coefficient was obtained 0.84. Also, its validity was obtained 0.58, using Spearman's correlation coefficient.

## Results

The patients’ mean age was 28.67±7.09. Fourteen patients were single and 16 married. Table 1 shows a comparison of the mean changes in gastrointestinal symptoms in the two groups at pre-test, post-test and one month follow-up.

**Table 1:** The mean and standard deviation of functional dyspepsia symptoms in the experimental and control groups in the pre-test, post-test and follow-up

**Groups**	Pre-test	Post-test	Follow-up
**Experimental**	53.66±23.09	12.66±12.26	4.93±7.12
**Control**	68.13±13.98	65.40±15.71	65.40±13.65
**p**	0.047	<0.001	<0.001

*Significant level: 0.05

The mean scores of dyspepsia in the pre-test, post-test and follow-up in the experimental group were 53.66, 20.86 and 18.13, respectively. Also, in the control group they 55.93, 57.80 and 56.53, were respectively (Table 1). These results show that symptoms of functional dyspepsia reduced in the post-test. Also, the results showed that the effectiveness of interventions was maintained at follow-up.

Levine test showed that there was not significant in difference in functional dyspepsia (F=0.224, p=0.64). Thus, there was no significant difference between experimental and control groups in variance in symptoms of functional dyspepsia; this confirms the assumption of homogeneity of variances. Considering the fact that in this study functional dyspepsia was the dependent variable, we used analysis of covariance with one variable for data analysis. 

**Table 2:** Results of homogeneity of regression slopes between covariates and dependent on operating level

Index	SS	df	MS	F	p
Pre-tests and post-tests of interaction	34.16	1	17.08	0.10	0.900

*Significant level: 0.05

There were no significant interaction between dependent covariates and factor levels. So, compliance the assumption of homogeneity of regression (Table 2).

**Table 3:** Results MANCOVA of mean scores functional dyspepsia in the experimental and control groups on the follow-up

Test	Mean difference	F	Sig
Post-test	52.73	84.73	<0.001
follow-up	60.46	197.15	<0.001

*Significant level: 0.05

There was a significant difference between the experimental and control groups in terms of functional dyspepsia on the post- test phase (F=84.73, p<0.000) (Table 3). Also, There was a significant difference between the experimental and control groups in terms of functional dyspepsia on the follow-up phase (F=197.15, p<0.000) (Table 3).

## Discussion

The purpose of this study was to investigate the effectiveness of cognitive-behavioral stress management on functional dyspepsia symptoms in patients who referred to digestive clinics in Ahvaz. Results of data analysis showed that cognitive behavioral stress management reduced the symptoms of functional dyspepsia in the experimental group patients compared with the control group. Also, this difference was sustained one month after the follow up. The present results are consistent with those of the studies conducted by Bagherian Sararudi et al. (2009) and Drossman (1995) (18, 23). Patients with functional dyspepsia did not support social relationships and self-esteem compared to those who had low self-esteem and social support were more likely to experience physical and psychological problems (24). Individuals with psychosocial resources are vulnerable to disease and mood disorders, particularly in response to stress. Providing social support for patients with dyspepsia might not only enable them to be successful against stressful events, but also affect the behaviors associated with stress. These findings suggested that such patients may benefit from psychological and psychiatric interventions. Drosman’s (1995) study showed that cognitive- behavioral therapy and stress management as interpersonal psychotherapy and relaxation techniques can reduce anxiety, pain and depression in gastrointestinal disorders. In order to explain the results of this research and the therapeutic efficacy, it can be said that stress management increases individual’s ability to reduce stress and adapt with stressful situations (23). This treatment is composed of elements such as raising awareness about stress, relaxation training, identification of dysfunctional thoughts, cognitive restructuring, problem solving training, expressiveness training, anger management, self-management and planning activities, all causing reduction in the functional dyspepsia symptoms. In patients with functional dyspepsia, the less they use problem-focused coping style and less seeking social support, the less they define the problem; they will be less able to find flexible solutions. Probably, depressive symptoms in patients with functional dyspepsia are due to lack of social support and problem-focused coping skills (24, 25). Providing social support for patients with functional dyspepsia will enable them to cope more successfully with stressful life events. Cognitive-behavioral stress management is an appropriate intervention that increases coping skills and social support in patients with functional dyspepsia. Stress management techniques have been applied successfully in many physical and emotional problems such as anxiety and depression, insomnia, fear of dental treatment, diabetes, high blood pressure, headaches, heart disease, genital herpes, arthritis, AIDS and cancer (26) and also functional syndromes such as irritable bowel syndrome and functional dyspepsia (27-29). Treatment using stress management improves the health status of patients. In this regard, the patients were instructed to identify the outcomes such as the upper abdominal pain, burning and other symptoms of functional dyspepsia and consequences this symptoms. They were asked to identify and avoid the stimulants arousing symptoms dyspepsia. Also, they gained more control over their symptoms by encouraging following the recommended diet.

Anger, worry and anxiety states are significant factors in stressed patients with functional dyspepsia and have a huge impact on the interpersonal relationships of patients with the disease. These patients are angry and irritable due to stress caused by bloating, upper abdominal irritation, nausea or vomiting and belching. This creates a cycle that moves between anxiety and symptoms of functional dyspepsia. Thus, anger management training and problem solving skills help the patients use appropriate methods for solving conflicts instead of violent reactions. Also, intervention within sessions was targeted to reduce symptoms through training various ways to challenge negative automatic thoughts and attitudes associated with patients with functional dyspepsia, encouraging patients to increase activity pleasurable and planning activities that lead to increased success in everyday life. 

## Conclusion

The results of the present study suggest that cognitive-behavioral stress management strategies are effective in reducing symptoms in patients with functional dyspepsia. So, cooperation among gastroenterologists, psychologists and psychiatrists can have positive effects on these patients. In general, it can be said that the relative reliability results of treatment at follow-up period are related to the use of actively therapeutic techniques provide by patients. The limitations of the present study were the high cost of diagnostic studies including sonography and endoscopy of the upper gastrointestinal system and the small sample size in this study. Also, the lack of a placebo group for further comparisons is another limitation of this study. According to the results, further studies are recommended to compare separate groups of men and women and evaluate the impact of gender on treatment effectiveness. The findings of this study can be used in therapeutic centers, counseling and gastrointestinal clinics, and by specialists of mental health clinics.  
